# Proteomic overview of hepatocellular carcinoma cell lines and generation of the spectral library

**DOI:** 10.1038/s41597-022-01845-x

**Published:** 2022-11-29

**Authors:** Mingchao Wang, Shuang Weng, Chaoying Li, Ying Jiang, Xiaohong Qian, Ping Xu, Wantao Ying

**Affiliations:** State Key Laboratory of Proteomics, Beijing Proteome Research Center, National Center for Protein Sciences (Beijing), Beijing institute of Lifeomics, Beijing, China

**Keywords:** Proteomics, Liver cancer

## Abstract

Cell lines are extensively used tools, therefore a comprehensive proteomic overview of hepatocellular carcinoma (HCC) cell lines and an extensive spectral library for data independent acquisition (DIA) quantification are necessary. Here, we present the proteome of nine commonly used HCC cell lines covering 9,208 protein groups, and the HCC spectral library containing 253,921 precursors, 168,811 peptides and 10,098 protein groups. The proteomic overview reveals the heterogeneity between different cell lines, and the similarity in proliferation and metastasis characteristics and drug targets-expression with tumour tissues. The HCC spectral library generating consumed 108 hours’ runtime for data dependent acquisition (DDA) of 48 runs, 24 hours’ runtime for database searching by MaxQuant version 2.0.3.0, and 1 hour’ runtime for processing by Spectronaut^TM^ version 15.2. The HCC spectral library supports quantification of 7,637 protein groups of triples 2-hour DIA analysis of HepG2 and discovering biological alteration. This study provides valuable resources for HCC cell lines and efficient DIA quantification on LC-Orbitrap platform, further help to explore the molecular mechanism and candidate therapeutic targets.

## Abstract (summary)

**Table:** 

Measurement(s)	Proteome of hepatocellular carcinoma cell lines
Technology Type(s)	Liquid chromatography-tandem mass spectrometry
Sample Characteristic - Organism	Homo sapiens

## Background & Summary

Liver cancer ranks the sixth most common cause of cancer-related death world widely^1^. Hepatocellular carcinoma (HCC) represents approximately 90% of all primary liver cancer^2^. Studies on the proteomic landscape of HCC have advanced our knowledge at the molecular basis. Based on the label-free proteomic data of hepatocellular carcinoma patients of BCLC 0-A stage, we defined three subtypes, and found SOAT1 as a potential therapeutic target^3^. Gao *et al*. identified the tumour characteristics in HCC patients by isobaric tandem mass tags (TMT)-based proteomics, and identified two prognostic biomarkers, PYCR2 and ADH1A^4^. Cancer cell lines are the most extensively used model systems in tumour biology and development of therapeutics^5^, thus, a clear understanding at the proteome level may help us make better usage of HCC cell lines to analyse molecular mechanism and screen anti-tumour drugs. In 2014, Megger, D.* et al*. analysed the proteome of mixture of HepG2, Hep3B and SK-Hep-1 by label-free analysis and identified 2,757 protein groups and 13,744 peptides^6^. In 2020, the proteome of 375 cell lines of the Cancer Cell Line Encyclopedia were analysed using TMT-based proteomics, while did not cover the commonly used HCC cell lines including HepG2.2.15, PLC/PRF/5, MHCC97L, MHCC97H, HCCLM3 and HCCLM6^7^. Recently, Goncalves, E. *et al*.^8^ identified 8,497 protein groups from 949 cell lines by data independent acquisition (DIA) method and identified 5,302 protein groups from Huh7 and 5,589 protein groups from Hep3B. However, whether HCC cell lines are identical or heterogeneous at proteome level was still not being revealed. Meanwhile, it remains unknown whether HCC cell lines are representative of primary HCC tumour at the proteome level. Thus, systemic exploration on the proteomic characteristics of HCC cell lines, and their comparison with primary HCC tumour is still necessary.

DIA mass spectrometry is an emerging method for quantifying protein groups consistently and accurately across multiple samples^9^. DIA quantification is based on the MS2 level through extraction of fragment ion chromatograms, which are less prone to be interfered than MS1 peak area^10^. DIA data can be analysed by the spectral library-based approach or the library-free approach. Both approaches could provide highly convergent identification and reliable quantification performance^11,12^. The spectral library is usually generated through data dependent acquisition (DDA) measurement of the peptides to be analysed by DIA^13^ and provides the precursor peptide-fragment connection^14^. Recently, it has been reported that the reproducibility, specificity, and accuracy of spectral library-based approach of DIA quantification is superior to DDA^12,15^. Thus, an HCC spectral library covering protein groups from HCC cell lines and primary tumour tissues could provide a valuable resource for DIA quantification, thus further support discovery of novel molecular mechanism and candidate therapeutic targets of HCC.

Here, we present the proteomic overview of nine commonly used HCC cell lines covering 9,208 protein groups, and an in-depth HCC spectral library containing 253,921 precursors, 168,811peptides (of which 150,327 peptides were proteotypic) and 10,098 protein groups. We revealed the poor consistency of proteome with transcriptome of these cell lines. Characteristic pathways of each cell line, and difference and similarity between HCC tissues were demonstrated. The HCC spectral library was used to analysis the differentially induced protein groups upon TGFB1 stimulation on HCCLM3 by Spectronaut^TM^ version 15.2 (Biognosys AG, Switzerland) and a free software suite, DIA-NN version 1.8. In summary, our results obtained proteome and outstretched pathway overview of commonly used nine HCC cell lines, provided a valuable guide for the usage of these cell lines. The HCC spectral library generated was available for in-depth DIA quantification of HCC cell lines and could help to explore the molecular mechanism and candidate therapeutic targets of HCC. Our research provides a pipeline composed of sample choice, peptide pre-fractionation, spectral library generation and DIA quantification, which is universal and can be used for DIA quantification study in other tumours.

## Methods

### Study design

**Fig. 1:**
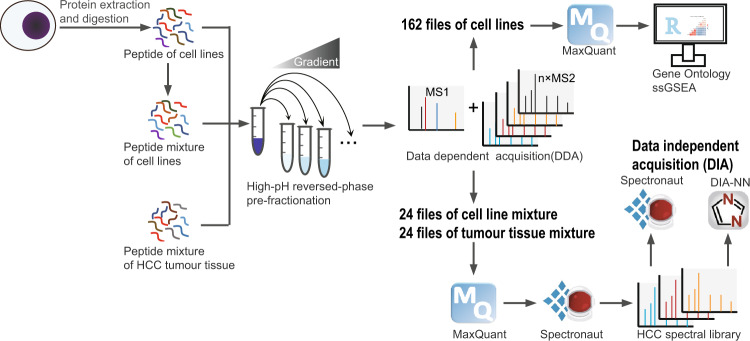
Workflow for the proteomic analysis of nine HCC cell lines and generation of the HCC spectral library. Nine HCC cell lines were protein extracted and trypsin digested. Peptides were pre-fractionated by Hp-RP and analysed by LC-MS/MS using DDA mode. The gained 162 raw files were searched again protein database by MaxQuant version 2.0.3.0. Gene Ontology (GO) and ssGSEA analysis were then implemented. For generation of the HCC spectral library, peptides of HCC cell line mixture or tumour tissue mixture were pre-fractionated, then analysed by LC-MS/MS using DDA mode. The gained 48 raw files were searched again protein database by MaxQuant version 2.0.3.0, and the search results were imported into Spectronaut^TM^ version 15.2 to generate the HCC spectral library. The HCC spectral library could be used in Spectronaut^TM^ version 15.2 and DIA-NN version 1.8 for DIA quantification.

Nine HCC cell lines were cultured, and then their total protein lysates were extracted and then trypsin digested to peptides, respectively. Peptides of each cell line was pre-fractionated by High-pH reversed-phase pre-fractionation (Hp-RP) to six fractions and analysed by liquid chromatography-tandem mass spectrometry (LC-MS/MS) using DDA mode (Table 1). The gained raw files were database searched by MaxQuant version 2.0.3.0. Gene Ontology (GO) and single sample gene set enrichment analysis^16^ (ssGSEA) were then implemented. For generation of the HCC spectral library, peptides of the HCC cell lines, or tumour tissues were mixed and fractionated by Hp-RP, respectively, and then analysed by LC-MS/MS (Table 1). The gained files were database searched by MaxQuant version 2.0.3.0, and the search results were imported into Spectronaut^TM^ (version 15.2, Biognosys AG, Switzerland) (Fig. 1) to generate spectral library which could be used in both Spectronaut^TM^ version 15.2 and DIA-NN version 1.8 for DIA quantification.

**Table 1:** Sample overview.

Sample name	Fractions	Number of repetitions	Number of raw files
HepG2	6	3	18
HepG2.2.15	6	3	18
Hep3B	6	3	18
Huh7	6	3	18
PLC/PRF/5	6	3	18
MHCC97L	6	3	18
MHCC97H	6	3	18
HCCLM3	6	3	18
HCCLM6	6	3	18
Cell line mixture	8	3	24
Tissue mixture	8	3	24

Sample names, numbers of fractions after High-pH reverse phase pre-fractionation and experimental repetition times.

### Cell culture

Cell lines were cultured in Dulbecco’s modified Eagle’s medium (DMEM, Corning, USA) containing 10% FBS (Gibco, USA), 100 U/mL penicillin and streptomycin mixture (Gibco, USA) in an incubator at 37 °C with 5% CO_2_. All cell lines we used were proven to be free from bacteria, fungi and mycoplasma by PCR. To investigate the effect of TGFB1 on HCCLM3, HCCLM3 was stimulated with 10 ng/mL of TGFB1 (R&D Systems, UK) for 48 hours.

### Protein extraction and digestion

Cell line samples were minced and lysed in Tissue Protein Extraction Reagent (T-PER, Thermo Scientific, USA) followed by 3 minutes of ultrasonic (1 second on and 2 second off, power 200 Watts) (SCIENTZT, JY92-II, China). The lysate was then centrifuged at 14,000 g for 15 minutes at 25 °C, and the supernatant was collected. The concentration of protein lysate was measured by the Bradford assay. The protein digestion was performed by filter-aided sample preparation (FASP)^17^. Each aliquot of 400 μg protein lysate was injected into a 30-kDa ultra-filter (Merck Millipore, Germany) followed by centrifugation at 14,000 g for 20 minutes at 25 °C. Then, 200 μL of UA solution (8 M Urea in 50 mM Tris-HCl, pH 8) with 10 mM DTT was injected into each ultra-filter. All the ultra-filters were kept for 2 hours at 37 °C for denaturing and reduction reaction. The solution in ultra-filters was removed by centrifugation at 14,000 g for 15 minutes at 25 °C, then 100 μL UA solution with 50 mM iodoacetamide (IAA, Sigma Aldrich, USA) was injected into each ultra-filter for alkylation. The ultra-filters were kept in dark for 30 minutes at 25 °C. After IAA incubation, the solution in ultra-filters was removed by centrifugation at 14,000 g for 10 minutes at 25 °C. Then, 200 μL of UA solution with 10 mM DTT was injected, and ultra-filters were kept at room temperature for another 15 minutes. The ultra-filters were centrifuged at 14,000 g for 15 minutes, and then washed with 200 μL UA solution once and 200 μL of ABC (25 mM ammonium bicarbonate, Sigma Aldrich, USA) three times by centrifugation at 14,000 g for 10 minutes at 25 °C. Then, 100 μL of ABC containing 8 μg trypsin (Promega, USA) was injected into each ultra-filter. All ultra-filters were incubated at 37 °C for 12 hours, and then peptide mixtures were collected into new collecting tubes by centrifugation at 14,000 g for 15 minutes at 25 °C. All ultra-filters were washed twice times with 100 μL of ABC by centrifugation at 14,000 g for 15 minutes at 25 °C. The flow-through solution was collected into the same collecting tube. The peptide concentration was measured using a Nanodrop 2000C (Thermo Scientific, USA) at 280-nm absorbance. The peptide mixtures were acidized with 10 μL of 4% trifluoroacetic acid (TFA, Sigma- Aldrich, USA), heat-dried and then stored at −80 °C.

### High-pH reversed-phase pre-fractionation

The peptide mixture was fractionated by Hp-RP with stepwise gradients manually. The C18 tip packed with 5 mg C18 reverse-phase media (3 μm, Durashell, Agela Technologies, China) was washed with 90 μL methanol (Sigma Aldrich, USA) and then with 90 μL ammonia water (pH 10). Then, 50 μg peptide re-dissolved in 160 μL ammonia water (pH 10) was loaded. And the tip was centrifuged at 1,000 g for 8 minutes at 25 °C to remove the liquid followed by washed with 90 μL of ammonia water (pH 10). Peptides binding on the C18 reverse-phase packing was then sequentially eluted with different concentration of acetonitrile (6%, 9%, 12%, 15%, 18%, 21%, 25%, 30%, and 50%) in ammonia water (pH 10). These fractions were collected, and the 25%, 30%, and 50% fractions were mixed with 6%, 9%, 12%, respectively. The final six fractions were heat-dried stored at −80 °C.

### LC-MS/MS analysis

For analysis of peptide mixture of each HCC cell line, the LC-MS/MS system consisted of a nanoflow high-performance liquid chromatograph (HPLC) instrument (EASY-nLC 1000 nanoflow LC, Thermo Scientific, USA) coupled to a Orbitrap Fusion Lumos Tribrid MS mass spectrometer (Thermo Scientific, USA) with a nano-electrospray ion source (Thermo Scientific, USA). For data acquisition, each fraction of peptide mixture was re-dissolved in mobile phase A (0.1% formic acid (FA, Sigma-Aldrich, USA), 99.9% pure water), and 1/10 of which was loaded onto the trapping column (100 μm × 20 mm, ReproSil-Pur C18-AQ, 3 μm; Dr Maisch, GmbH, Germany) using mobile phase A and then separated on the analytical column (150 μm × 150 mm, ReproSil-Pur C18-AQ, 1.9 μm; Dr Maisch, GmbH, Germany) at a flow rate of 320 nL/min with following gradients: 0–8 min, 5–8% mobile phase B (0.1% FA in 99.9% acetonitrile); 8–58 min, 8–23% mobile phase B; 58–70 min, 23–32% mobile phase B; 70–71 min, 32–95% mobile phase B; and 71–80 min, 95% mobile phase B. The Orbitrap Fusion Lumos was set to the OT-IT mode. For the MS1 scan, the AGC target was 5 × 10^5^ and the scan ranged from 300 to 1,400 m/z at a resolution of 120,000 and a maximum injection time of 50 ms. For the MS2 scan, a duty cycle of 3 s was set with the top-speed mode. Only spectra with a charge state of 2–6 were selected for fragmentation by higher-energy collision dissociation with a normalized collision energy of 35%. The MS2 spectra were acquired in the ion trap in rapid mode with an AGC target of 5,000 and a maximum injection time of 35 ms.

For analysis of peptides of HCCLM3 and TGFB1-stimulated HCCLM3 using DIA, the LC-MS/MS system consisted of the EASY-nLC 1000 nanoflow LC (Thermo Scientific, USA) coupled to the Q-Exactive HF mass spectrometer (Thermo Scientific, USA). For data acquisition, 2 μg peptide mixtures re-dissolved in mobile phase A was loaded and separated on the analytical column at a flow rate of 500 nL/min with following gradients: 0~13 min, 6 ~ 10% mobile phase B (0.1% FA in 99.9% acetonitrile); 13 ~ 99 min, 10~23% mobile phase B; 99 ~ 120 min, 23 ~ 33% mobile phase B; 120~123 min, 33 ~ 90% mobile phase B; 123 ~135 min, 90% mobile phase B. For the MS1 scan, the AGC target value was set to 3E6, and the m/z scan ranged from 400 to 1,200 Da at a resolution of 120,000 and a maximum injection time of 80 ms. For the MS2 scan, the isolation window range was set to 26 m/z at resolution of 30,000, and the AGC target was set to 5 × 10^5^. The maximum injection time for MS2 was set to auto. The normalized collision energy was set to 27, and the spectrum type was set to profile.

### Database searching for DDA and DIA raw files

The DDA raw files were searched against the human UniProt database (updated at 2022-09-07 containing 20,398 protein groups and the iRT peptide sequence) with MaxQuant version 2.0.3.0. The digestion mode was set to specific, and trypsin/P was chosen. Oxidation of methionine and acetylation of N-term of peptides were set as variable modification, and Carbamidomethyl of cysteine was set as fixed modification. False discovery rate (FDR) was set to 0.01 on both PSM and protein groups level. The max peptide mass range was set to 4,600 Da, and the peptide length range was set from 7 to 25, and the missed cleavage was set to 2. The MS/MS match tolerance was set to 20 ppm, and MS/MS de novo tolerance was set to 10 ppm. The proteinGroups.txt file generated by MaxQuant version 2.0.3.0 was then imported into Perseus v1.5.2.6 to extract the iBAQ value of each protein group of each cell line.

The DIA raw files were analysed by Spectronaut^TM^ version 15.2 against the HCC spectral library. Trypsin/P was chosen for digestion. Maximum intensity was used for intensity extraction of MS1 and MS2. Both correction factor for MS1 and MS2 mass tolerance were set to 1. XIC RT extraction window was set to dynamic and correction factor was set to 1. The calibration mode was set to automatic, and the iRT-RT regression was set to local (non-linear regression), and used Biognosys iRT Kit was chosen. Decoy method was set to Mutated, and decoy limit strategy were set to dynamic. Kernel density estimator was chosen to estimated p value. The precursor and protein group q-value cut-off was both set to 0.01. Proteotypic sequences and the MS2-level peak area were used for protein quantification and the same human UniProt database used for MaxQuant version 2.0.3.0 was set as the reference database. The top 3 precursors were used for peptide quantification, and the top 3 peptides were used for protein quantification. For DIA-NN version 1.8, Trypsin/P was chosen for digestion, and the miss cleavage site number was set to 2, and modifications including oxidation of methionine, acetylation of N-term and Carbamidomethyl of cysteine were chosen. Peptide length range was set to 7 to 25, and precursor charge range was set to 1 to 4, and precursor m/z range was set to 400 to 1,200, and fragment ion m/z was set to 200 to 2,000. The generated HCC spectral library was used as spectral. Single-pass mode neural network was chosen, and high accuracy was selected for quantification. RT-dependent cross-run normalization was selected. Precursor FDR was set to 1%.

### Generation of the HCC spectral library

Peptide mixture of cell lines and HCC tumour tissues was fractionated by Hp-RP with stepwise gradients manually. The C18 tip packed with 5 mg C18 reverse-phase media (3 μm, Durashell, Agela Technologies, China) was washed with 90 μL methanol (Sigma Aldrich, USA) and then with 90 μL ammonia water (pH 10). Then, 50 μg peptides dissolved in 160 μL ammonia water (pH 10) was loaded. And the tip was centrifuged at 1,000 g for 8 min at 25 °C to remove the liquid followed by washed with 90 μL of ammonia water (pH 10). Peptides were then sequentially eluted with 8 different concentrations of acetonitrile (9%, 12%, 15%, 18%, 21%, 25%, 30%, and 50%) in ammonia water (pH 10). These fractions were collected, heat-dried and stored at −80 °C.

The LC-MS/MS detection system consisted of the EASY-nLC 1000 nanoflow LC (Thermo Scientific, USA)coupled to the Q-Exactive HF mass spectrometer (Thermo Scientific, USA). For data acquisition, 1/8 of each of the Hp-RP fractions re-dissolved in mobile phase A was loaded and separated with the analytical column at a flow rate of 500 nL/min with following gradients: 0~13 min, 7 ~ 13% mobile phase B (0.1% FA in 99.9% acetonitrile); 13 ~ 99 min, 13~28% mobile phase B; 99 ~ 120 min, 28 ~ 42% mobile phase B; 120~123 min, 42 ~ 95% mobile phase B; 123 ~135 min, 95% mobile phase B. For the MS1 scan, the target value was set to 3E6 and the m/z scan ranged from 300 to 1,400 Da at a resolution of 120,000 and a maximum injection time of 80 ms. Only spectra with charge states of 2~ 6 were selected for fragmentation with a normalized collision energy of 27%. Precursor ions with top 20 intensities were selected for fragmentation. For the MS2 scan, the AGC target value was 5E4 and the resolution was 15,000 with a maximum injection time of 45 ms. The iRT peptide standards (Biognosys AG, Schlieren-Zürich, Switzerland) were spiked into all runs of spectral library generation.

The obtained DDA raw files were searched against the human UniProt database (updated at 2022-09-07 with 20,398 protein groups and the iRT peptide sequence) by MaxQuant version 2.0.3.0. The digestion mode was set to specific, and trypsin/P was chosen. Oxidation of methionine and acetylation of N-term of peptides were set as variable modification, and Carbamidomethyl of cysteine was set as fixed modification. FDR was set to 0.01 on both PSM and protein groups level. The max peptide mass range was set to 4,600 Da, and the peptide length range was set from 7 to 25, and the missed cleavage was set to 2. The MS/MS match tolerance was set to 20 ppm, and MS/MS de novo tolerance was set to 10 ppm.

The search results of MaxQuant version 2.0.3.0 were imported into Spectronaut^TM^ version 15.2 to generate the HCC spectral library. The missed cleavage site number for peptide was set to 2. The m/z range was set as 400 to 1,200 Da, and the best N fragments per peptide was set as 3 to 6. B and y fragments were chosen, and modifications including oxidation of methionine, acetylation of N-term of peptides and Carbamidomethyl of cysteine was kept during library generation. The empirical iRT database was set as the iRT reference, and the minimum square cutoff was set to 0.8. FDR was set to 0.01 on both precursor and protein level. For calibration and main search, the tolerance was set to dynamic.

### Data processing

The R package NormalyzerDE (v1.8.0)^18^ was used for data normalization and quantile function was used. Differently expressed protein groups between cell lines or tissues were identified by R package limma (v3.46.0)^19^. GO analysis was implemented using R package clusterProfiler (v3.18.1)^20^ using enricher function. The ssGSEA analysis was implemented using R package GSVA (v3.18.2)^21^ using GSVA function and ssGSEA method. Gene sets recorded in the Molecular Signatures Database (MSigDB) v7.5.1^22^ was used as reference gene sets for all the analysis. Protein groups whose fold-change value was greater than 1.5 and adjust p-value was less than 0.01 was chosen as differently expressed protein groups. All the analysis was operated on R software (v4.0.3).

## Data Records

The 162 DDA raw mass spectrometry data (.raw) had been deposited to the ProteomeXchange Consortium via PRIDE^23^ with the dataset identifier PXD036643^24^. The 48 DDA raw mass spectrometry data (.raw) for library generation had been deposited to the ProteomeXchange Consortium via PRIDE with the dataset identifier PXD035028^25^.

The HCC spectral library at Figshare^26^ and QC reports generated by DIALib-QC, the search result of DDA raw files of HCC cell lines and spectral library generation by MaxQuant version 2.0.3.0, DIA raw mass spectrometry data (.raw) and all search results of HepG2, HCCLM3 and TGFB1 stimulated HCCLM3 by DIA-NN version 1.8 and Spectronaut^TM^ version 15.2 had been deposited to the ProteomeXchange Consortium via PRIDE with the dataset identifier PXD037159^27^.

## Technical Validation

### Proteomic profiling of HCC cell lines

**Fig. 2:**
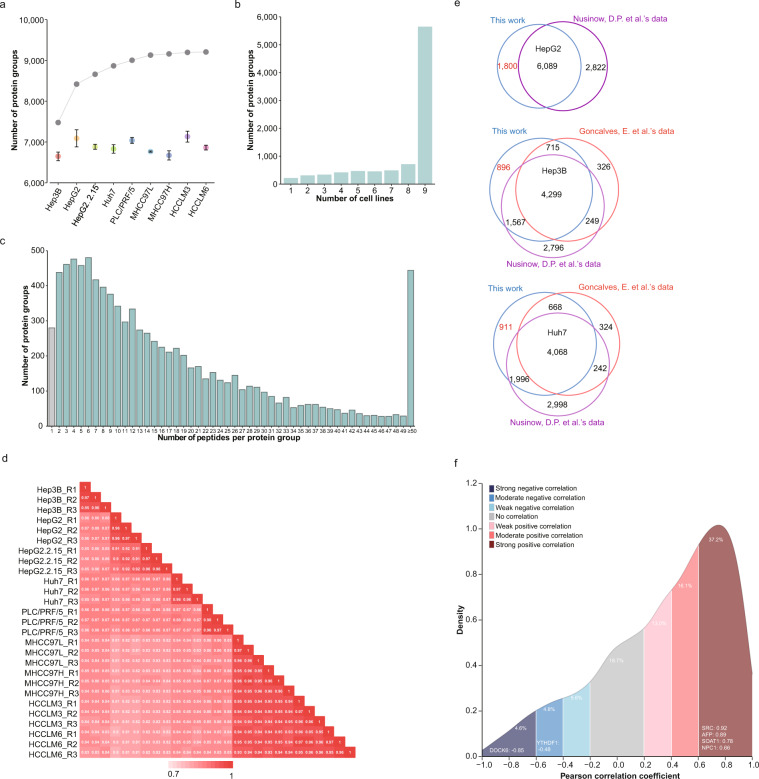
Proteome profiling of HCC cell lines. (**a**) The identified protein group number of nine HCC cell lines. The cumulative curve was shown on the top in gray. (**b**) Bar plot showed the number of protein groups detected in different number of HCC cell lines. (**c**) Bar plot showed the number of protein groups having different number of peptides. (**d**) Heatmap showed the Pearson correlation coefficients of nine HCC cell lines. (**e**) Venn diagrams shows the uniquely identified protein groups in this study compared with data reported by Nusinow, D.P. *et al*.^7^ and Gonçalves, E. *et al.*^8^. (**f**) Density plot shows the proteome-transcriptome correlation distribution of protein groups by five HCC cell lines. Different Pearson correlation coefficient ranges are represented by different colours. Representative proteins are marked in the figure.

Cumulatively, 9,208 protein groups were identified from 162 peptide fractions of nine HCC cell lines, and the average identified number was 7,699 (Fig. 2a), and 61.5% (5,664 of 9,208) were all detected in the nine HCC cell lines (Fig. 2b). These protein groups correspond to 160,042 peptides, and 97% (8,928 of 9,208) of protein groups had at least two unique peptides (Fig. 2c). Three repetitions of each cell lines have high quantitative repeatability (Pearson correlation coefficient > = 0.95 between three repetitions of each cell line, Fig. 2d). All HCC cell lines showed similarity with others (Pearson correlation coefficient > = 0.8), and MHCC97L, MHCC97H, HCCLM3 and HCCLM6 showed high consistency with each other (Pearson correlation coefficient > = 0.93), and HepG2 showed a high consistency with HepG2.2.15 (Pearson correlation coefficient > = 0.9). Compared with the data reported by Goncalves, E. *et al*. and Nusinow, D.P. *et al*., our proteomic data still uniquely identified 1,800, 896 and 911 protein groups from HepG2, Hep3B and Huh7 (Fig. 2e).

A poor correlation between the proteome and transcriptome was always revealed in cell lines^7^ and human tissues^28^.Comparison of the transcriptome with proteome of five HCC cell lines (Hep3B, HepG2, Huh7, MHCC97H and PLC/PRF/5 in GSE97098^29^, we also revealed a poor consistency between the proteome and transcriptome: the mean of Pearson correlation coefficient was 0.34; 37.2% of protein groups showed high consistency (Pearson correlation coefficient > 0.6) with their transcriptome, including SOAT1 and NPC1, two core molecules for cholesterol metabolism^30^, and AFP, an important biomarker of HCC^31^, and SRC, an important tyrosine protein kinase for cancer proliferation and metastasis^32^. However, we also found that 9.4% of protein groups showed negative correlation (Pearson correlation coefficient < −0.4) with their transcriptome, including DOCK6, a molecule which could promote chemo- and radio-resistance in cancer^33^, and YTHDF1, a key regulator of m^6^A methylation^34^ (Fig. 2f). This poor correlation maybe due to the differences of normalization strategies between proteome and transcriptome, and also may cause by multiple biological factors including mRNA degradation rate, ribosome binding rate, ribosome density, codon usage bias, protein turnover, PTM variants, peptide sharing among isoforms, low abundant protein and experimental noises^35^. The existing inconsistency between proteome and transcriptome highlighted the necessity of a proteomic overview of these cell lines.

### Proteomic characteristics of HCC cell lines

**Fig. 3:**
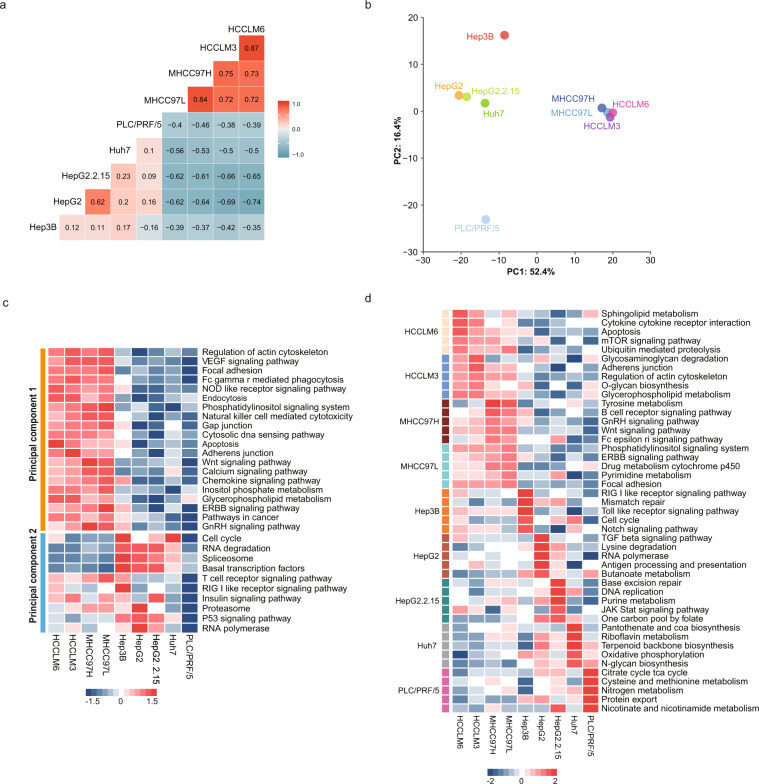
The proteome-based pathway overview of HCC cell lines. (**a**) Pearson correlation coefficients of pathway ssGSEA score of HCC cell lines. (**b**) The principal component analysis results are showed on the two-dimensional plane composed of principal component 1 and principal component 2. Nine HCC cell lines were represented by different colours. (**c**) Heatmap shows the normalized ssGSEA scores of pathway alteration on principal component 1 and principal component 2. (**d**) Heatmap shows the normalized ssGSEA scores of uniquely enriched pathways in each HCC cell line.

High consistency was revealed among MHCC97L, MHCC97H, HCCLM3 and HCCLM6, and between HepG2 and HepG2.2.15 according their ssGSEA score of pathways (Fig. 3a). These are in good agreement with backgrounds of these cell lines: MHCC97L, MHCC97H, HCCLM3 and HCCLM6 were derived from the same progenitor cell line, MHCC97^36^, and HepG2.2.15 was derived from HepG2 and characterized by stable HBV DNA^37^. Principal component analysis based on ssGSEA score of pathways were showed on the two-dimensional plane composed of principal component 1 (52.4%) and principal component 2 (16.4%): MHCC97L, MHCC97H, HCCLM3 and HCCLM6 almost overlapped, and were far from other cell lines of principal component 1; Hep3B and PLC/PRF/5 displayed the maximum distance of principal component 2 (Fig. 3b). Pathways including actin cytoskeleton, VEGF signalling pathway, focal adhesion were highly variable on principal component 1, and cell cycle, RNA degradation and spliceosome on principal component 2 (Fig. 3c Furthermore, we found that each cell line has its uniquely enriched pathways. Cancer-related pathways such as Wnt signalling pathway^38^, cell cycle^39^ and TGF beta signalling pathway^40^ were heterogeneously enriched in different HCC cell lines (Fig. 3d). It is meaningful to consider these heterogeneities before building of cell models for targets validation.

### Cancer cell lines retain tumour characteristics of HCC tissues

**Fig. 4:**
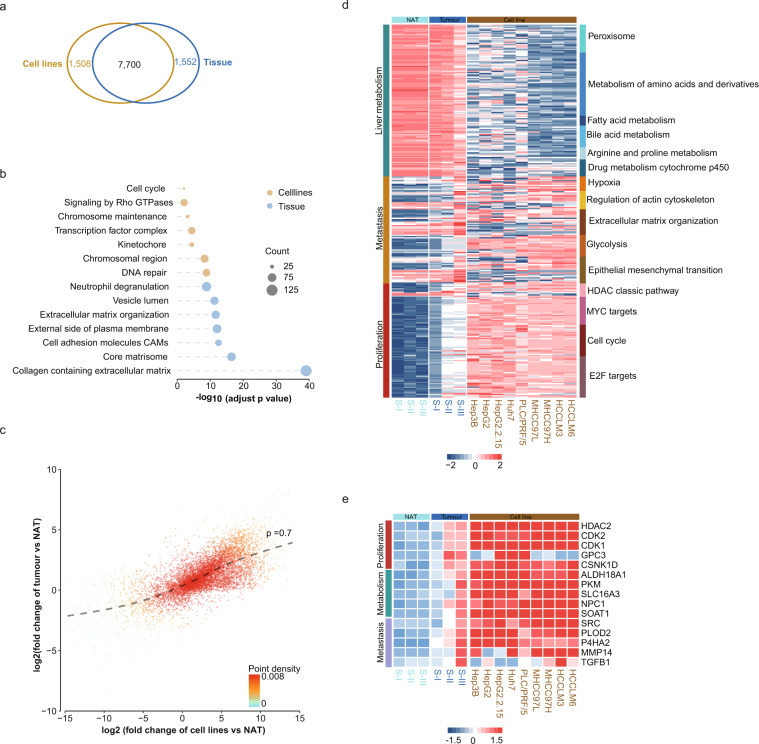
Molecular characteristics of HCC cell lines relative to tumour and NAT. (**a**) Venn diagram shows number of protein groups identified only in cell lines (orange), only in tissue (blue) and both in cell lines and tissue (black). (**b**) Go enrichment of proteins identified only in cell lines (orange), or only in tissue (blue). (**c**) The consistency between log2 transformed fold change between tumour tissue versus NAT and cell lines versus NAT. (**d**) Heatmap shows the normalized abundance of proteins in representative cancer-related pathways. (**e**) The protein abundance of drug targets expressed in both tissue and HCC cell lines.

We found that the 1,508 protein groups only expressed in HCC cell lines were enriched in cell cycle, signalling by Rho GTPases, kinetochore, chromosome and DNA repair, while the 1,552 protein groups uniquely expressed in tissue were enriched in extracellular matrix, complement and blood, indicated that one main difference between cultured HCC cell lines with tissue is the deficiency of extracellular microenvironment (Fig. 4a,b). HCC cell lines and tumour tissues exhibited a high correlation of expression change relative to normal adjacent tissues (NAT) (Pearson’s correlation coefficient = 0.7, Fig. 4c). Detailed pathway enrichment analysis revealed that all HCC cell lines retained the proliferation and metastasis of HCC, meanwhile MCC97L, MHCC97H, HCCLM3 and HCCLM6 totally lose the liver metabolism related function, indicated that HCC cell lines could be considered as more oncological subtypes of HCC (Fig. 4d). Jiang *et al*.^3^ found 21 candidate drug targets, 15 of which were detected in these cell lines, but with different expression characteristics: drug targets involving proliferation including HDAC2, CDK1, CDK2, CSNK1D were highly expressed in all the nine HCC cell lines, while GPC3 only detected in HepG2.2.15, Huh7 and PLC/PRF/5. Drug targets involving metabolism including ALDHA8A1, PKM, SLC16A3, NPC1 and SOAT1 were highly expressed in all the nine HCC cell lines. Drug targets involving metastasis including SRC, PLOD2 and P4HA2 were also detected in all the HCC nine cell lines, while MMP14 only showed low expression in HepG2 and HepG2.2.15, and TGFB1 showed highest expression in HCCLM3 (Fig. 4e).

### Properties of HCC spectral library

**Fig. 5:**
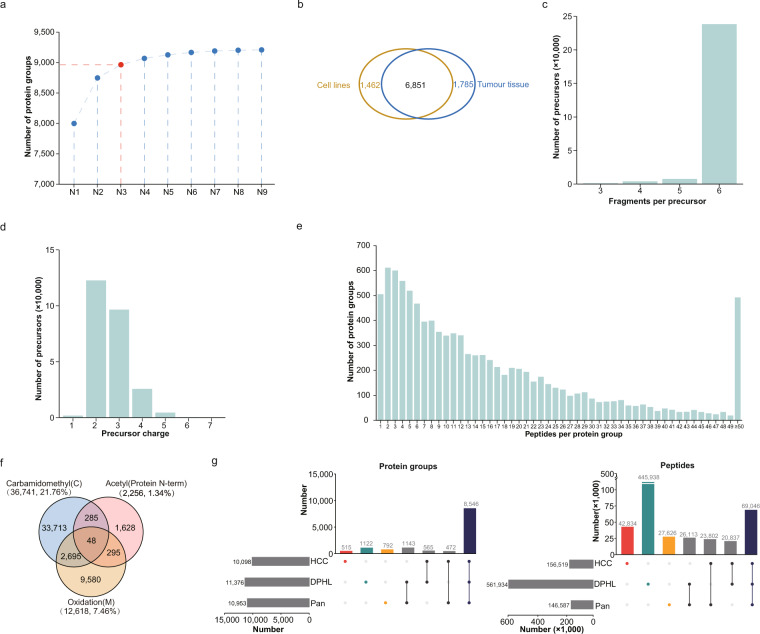
Overview of the HCC spectral library. (**a**) The max identified number of protein groups of different combinations of HCC cell lines. From N1 to N9 means the combinations of different number (from one to nine) of cell lines. Combining of the identified protein number of three cell lines HCCLM3, HepG2 and PLC/PRF/5 could cover 97% (8,964 of 9,208) of the protein groups identified in nine HCC cell lines, and this combine was coloured by red in the picture. (**b**) Number of protein groups in the HCC spectral library identified from HCC cell lines (orange) or tumour tissue (blue). (**c**) Bar plot showed the number of precursors by the number of fragments per precursor. (**d**) Bar plot showed the number of precursors by the charge states of precursor. (**e**) Bar plot showed the number of protein groups by the number of peptides per protein group. (**f**) Venn diagram shows number of peptides with different modifications. (**g**) Comparison the covered protein groups and peptides of the HCC spectral library with Pan Human library^42^ and DPHL library^43^. Protein groups and peptides uniquely covered in the HCC spectral library was coloured by red.

We generated an HCC spectral library covering protein groups from HCC cell lines and tumour tissue, to support the DIA quantification. We calculated the covered protein groups number of combination of different number (from 2 to 8) of nine HCC cell lines. Then, for combinations with a specific number, we select the combination with the max covered protein groups number. We found that the covered protein groups number of combination three HCC cell lines, HCCLM3, HepG2 and PLC/PRF/5, could cover 97% (8,964 of 9,208) of protein groups covered by all the nine HCC cell lines (Fig. 5a). Thus, peptides of HCCLM3, HepG2 and PLC/PRF/5 were mixed and used for spectral library generation. The peptide mixture of HCC tumour tissue^3^ was also used for spectral library generation. The generated HCC spectral library at Figshare^26^ covered 253,921 precursors, 168,811 modified peptides (156,519 peptides, of which 150,327 peptides were proteotypic) and 10,098 protein groups. Evaluation by DIALib-QC^41^ showed a high quality of the HCC spectral library. About 14.5% (1,462 of 10,098) protein groups were exclusively provided by DDA files of HCC cell lines, while 17.7% (1,775 of 10,098) provided by tumour tissue DDA files only (Fig. 5b). About 94% (238,930 of 253,921) of the precursors have 6 fragment ions, as we set the best N fragments per peptide was set as 3 to 6 (Fig. 5c). Precursor charge states range from + 1 to + 7, in which 97% (246,004 of 253,921) are of charge states between + 2 and + 4 (Fig. 5d). Protein groups with more than 2 unique peptides per protein group constitute about 95% (9,591 of 10,098) of the protein groups in the spectral library (Fig. 5e). Statistics of post translation modifications found that 36,741 (21.76%) peptides have carbamidomethyl modification, and 2,256 (1.34%) peptides have acetyl modification on the N-term of protein, and 12,618(7.46%) peptides have oxidation modification on methionine residue (Fig. 5f). Compared with the reported Pan human library^42^ and DPHL library^43^, we found that the HCC spectral library uniquely covered 515 protein groups and 42,834 peptides (Fig. 5g).

### Applicability of the HCC spectral library for DIA analysis

**Fig. 6:**
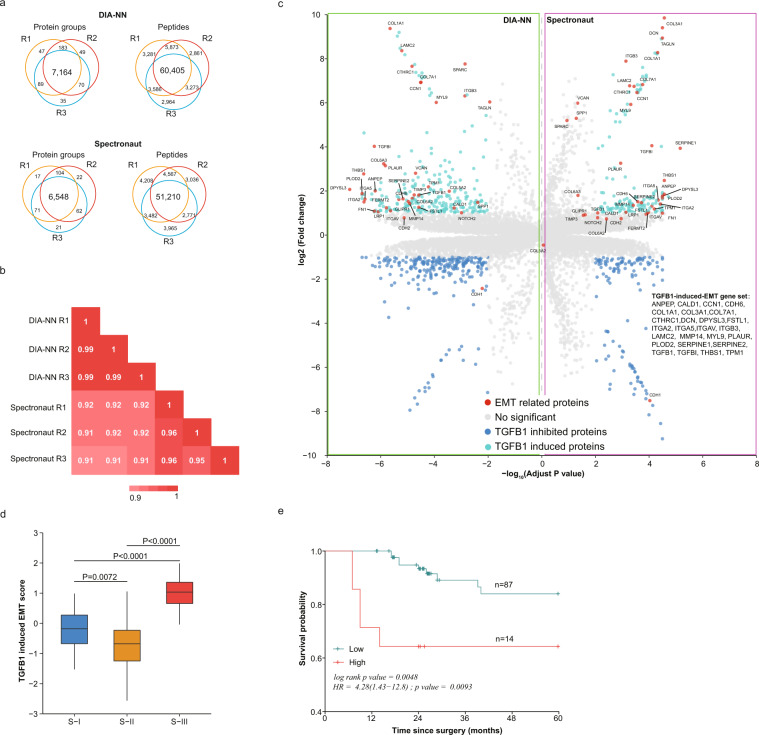
Performance of the HCC spectral library on DIA quantification. (**a**) Venn diagram showed the overlap of identified protein groups and peptides of HepG2 in DIA quantification by DIA-NN version 1.8 and Spectronaut^TM^ version 15.2. R1, R2, R3 represents three independent repeated experiments. (**b**) Heatmap showed the Pearson correlation coefficients of protein abundance between DIA-NN version 1.8 and Spectronaut^TM^ version 15.2. R1, R2, R3 represents three independent repeated experiments. (**c**) Volcano plot shows the differently expressed protein groups between HCCLM3 and TGFB1-induced HCCLM3 identified by DIA-NN version 1.8 and Spectronaut^TM^ version 15.2 based on the HCC spectral library. Proteins up-regulated after TGFB1 stimulating was represented by green, and down-regulated proteins by blue. The EMT related proteins was labelled by black font and coloured by red. (**d**) Boxplots showed the distribution of TGFB1-EMT score in early HCC subtypes. In the boxplots, the middle bar represents the median, and the box represents the interquartile range; bars extend to 1.5 × the interquartile range. (S-I, blue; S-II, orange; S-III, red). The P values of Wilcox test were labelled on the top. (**e**) Curves shown the five-year overall survival of patients in TGFB1-EMT high (n = 14, red) and low group (n = 87, green). The p value for log-rank test, the HR and its 95% confidence interval (HR (95%CI)) and p value were labelled on the picture.

We could identify 7,637 protein groups and 82,243 peptides in triples 2-hour DIA analysis of HepG2 peptides using the HCC spectral library and DIA-NN version 1.8 with optimized parameters for LC-MS/MS^44^, and 94.2% (7,194 of 7,637) of protein groups and 73.4% (60,405 of 82,243) of peptides were quantified three times. Meanwhile, 6,845 protein groups and 73,599 peptides could be identified from the same raw files by Spectronaut^TM^ version 15.2, and 95.7% (6,548 of 6,845) of protein groups and 69.6% (51,210 of 73,599) were quantified three times (Fig. 6a). High quantitative reproducibility (Pearson correlation coefficient >0.9, Fig. 6b) was revealed between repeated experiments analysed with DIA-NN version 1.8 or Spectronaut^TM^ version 15.2. We then analysed an experimental mode driven from HCCLM3 (TGFB1 stimulated HCCLM3 vs control). We observed down-regulation of CDH1, the main initiation signals of EMT^45^, and up-regulation of THBS1^46^ and CDH6^47^, two proteins whose up-regulation could represent the activation of EMT by both DIA-NN version 1.8 and Spectronaut^TM^ version 15.2 (Fig. 6c). In 121 protein groups identified as TGFB1-induced up-regulated proteins, 26 were annotated as members of EMT hallmark by Molecular Signatures Database v7.5.1, and they were further defined as the TGFB1-induced-EMT gene set (Fig. 6c). Based on this gene set, we calculated the TGFB1-EMT score of each patient in Jiang *et al*.’s cohort^3^ using ssGSEA algorithm. The TGFB1-EMT score of patients of S-III tumour was significantly (P < 0.0001) higher than S-I or S-II (Fig. 6d). The 101 patients could be stratified into TGFB1-EMT-high (n = 14) and TGFB1-EMT-low (n = 87) group according to their TGFB1- EMT score. The five-year overall survival rate of TGFB1-EMT-high group was significantly lower than the TGFB1-EMT-low group (overall survival rate: 64.3% (95%CI: 43.5%~95.0%) vs 84.0% (95%CI: 74.4%~94.8%), log-rank P value = 0.0048; the hazard ratio (HR of TGFB1-EMT-high group vs TGFB1-EMT-low group was 4.28 (95% CI: 1.43~12.8), P value = 0.0093) (Fig. 6e). These results indicated that TGFB1-induced EMT is closely related to poor prognosis of early HCC patients, and the novel defined TGFB1-induced-EMT gene set maybe useful for predict the prognosis of early HCC patients.

## Data Availability

No custom computer codes were generated in this work.
